# Dataset on organizational innovation and its determinants in the SMEs hotels

**DOI:** 10.1016/j.dib.2019.104352

**Published:** 2019-08-10

**Authors:** Nestor Montalvan-Burbano, Jose Antonio Plaza-Ubeda, Miguel Perez-Valls, David Sabando-Vera

**Affiliations:** a University of Almería, Ctra. Sacramento s/n, La Cañada de San Urbano, 04120, Almería, Spain; b Espol Polytechnic University, Campus Gustavo Galindo Km. 30.5 Vía Perimetral, Guayaquil, Ecuador

**Keywords:** Organizational innovation, Managerial innovation, SMEs, Hotels, Ecuador

## Abstract

The aim of the study is to identify the factors that influence innovation activities associated with business management, known in the academic world as organizational innovation. Data was gathered by administering a survey on the managers or owners of hotels in the province of Santa Elena, Ecuador. Three components of organizational innovation were analyzed: methods of organizing job positions, work organization practices and management of external relations; all of which were tested with both internal variables (individual and structural characteristics) and variables external to the firms.

**Table:** Specifications Table

Subject area	*Business, Management and Accounting*
More specific subject area	*Business and Organizational Innovation*
Type of data	*Table, Figure*
How data was acquired	*Through questionnaire to managers or owners of hotels*
Data format	*Raw*
Experimental factors	- *The population is comprised of the hotels registered with the Provincial Department of Tourism – the institution which issues operation permits*. - *The data set allows a rational evaluation of organizational innovation in the administrative management of a firm*.
Experimental features	*The researchers established a sample of 146 hotels and surveyed key informants of the firm's administration with a semi-structured survey*.
Data source location	*Province of Santa Elena, Ecuador*
Data accessibility	*Data is with the article*

**Table:** 

**Value of the Data** • The data set makes it possible to identify how the methods of organizing job positions, work organization practices and the management of external relations are modified by internal and external factors associated with business management. • The data can be used by researchers in the field and managers to better understand the potential benefits of comprehending business management from the perspective of organizational innovation. • The data provide a baseline for further study of organizational innovation in the context of hotels or SMEs, as well as for future evaluation of the different variables for the purpose of producing critical analyses.

## 1 Data

The data correspond to quantitative research of the hotel sector in the context of small and medium enterprises. The research obtained information from the owners and/or managers of the businesses to identify the factors that influence this business activity, with the aim of implementing new administrative and organizational structures and management practices that enable enterprises to create value and achieve their proposed objectives [1], [2]. These new structures and practices together are known as Organizational Innovation, a term that is relatively recent and synonymous with other terms such as Management Innovation o Administrative Innovation [3].

Organizational innovation allows the company to generate innovation, creativity, competitive advantage and improve its performance [4], [5], [6]. Despite its relevance in the business context, there are not many works regarding the factor related to organizational innovation, especially in the hotel sector [7].

In this context, the studies of the hotel industry are focused on Europe, Asia, and North America, being scarcely explored in developing countries [8]. For this purpose, data were collected from the hotel sector of Ecuador as an academic contribution in the field of Organizational Innovation in small and medium enterprises (SMEs). The dataset (Appendix A) exhibits information on the determinants of organizational innovation in micro firms (89.7%), small firms (9.6%) and medium firms (0.7%), with more than five years in operation in 68.5% of cases. Table 1 shows basic information about the characteristics of the companies dataset.

**Table 1:** Enterprises basic information of the dataset.

Parameter	Characteristics	Number of Firms	Firms (In percent)
Size	Micro firm	131	89.70%
	Small firm	14	9.60%
	Medium firm	1	0.70%
Type	Special taxpayer	3	2.10%
	Limited company	1	0.70%
	Natural person obligated to keep accounting	83	56.80%
	Natural person not obligated to keep accounting	49	33.60%
Age	Less than 5 years	46	31.50%
	More than 5 years	100	68.50%
Observations		146	100%

For a better understanding of the data it is necessary to make some clarifications:

• Organizational Innovation is related to the administration and its commitment to renew at the organizational level systems, procedures, and techniques in order to obtain information exchange leading to collaboration, learning, and innovation [1], [9].
• The company is represented by the owner, general manager or administrative assistant, who are considered informants.

Table 2, Table 3, shows the variables of the data. The variables represent both internal and external factors related to organizational innovation, as well as some basic elements related to the size and age of the firm. Researchers interested in the development of organizational innovation, linking two scarcely studied areas such as SMEs and the hotel industry, can use the variables and the data set.

**Table 2:** Variables of the dataset: Internal and Organizational Innovation Factors.

Field	Variable	Question Type	Labels value
TYPE_1	Company type	Single choice	1 - Natural person not obligated to keep accounting, 2 - Natural person obligated to keep accounting, 3 - Limited company, 4 - Anonymous society, 5 - Special taxpayer,
AGE_1	Age of company	Single choice	1 - Less than 5 years, 2 - More than 5 years
SIZE_1	size of the company	Single choice	1 - Micro Firm (less than 10 employees), 2 - Small Firm (10–50 employees), 3 - Medium Firm (51–250 employees)
NET_1	Network of collaboration	Single choice	0 - No, 1 - Yes
TRA_1	Administration position of the interview	Single choice	1 - Administrative assistant, 2 - Owner, 3 - General Manager - 4 - Manager Owner
TRA_2	Work experience of the interviewee	Single choice	1 - Less than 1 year, 2–1–2 years, 3 - 3–4 years, 4–5 or more years
TRA_3	Degree of education of the interviewee	Single choice	1 - Elemental education, 2 - High School, 3- technological/technical, 4 - University degree 5 - master's degree
OI_1	New practices in the organization	Single choice	0 - No, 1 - Yes
OI_2	Organization new methods	Single choice	0 - No, 1 - Yes
OI_3	Management new methods	Single choice	0 - No, 1 - Yes

**Table 3:** Variables of the dataset: External Factors of Organizational Innovation.

Field	Variable	Question Type	Labels value
OA_1	answer to needs of a client	Single choice	1 -Null, 2- Low, 3 - Medium, 4 - High
OA_2	Improvement of the ability to develop new processes	Single choice	1 -Null, 2- Low, 3 - Medium, 4 - High
OA_3	Higher quality of its services	Single choice	1 -Null, 2- Low, 3 - Medium, 4 - High
OA_4	Lower costs per unit of production of the service	Single choice	1 -Null, 2- Low, 3 - Medium, 4 - High
OA_5	Improvement in the exchange of information or communication within the company	Single choice	1 -Null, 2- Low, 3 - Medium, 4 - High
IB_1	Cost Factors - Lack of funds in the company	Single choice	1 -Null, 2- Low, 3 - Medium, 4 - High
IB_2	Cost Factors - Lack of financing for the company	Single choice	1 -Null, 2- Low, 3 - Medium, 4 - High
IB_3	Cost Factors - Innovation has a high cost	Single choice	1 -Null, 2- Low, 3 - Medium, 4 - High
IB_4	Knowledge Factors - Lack of qualified personnel	Single choice	1 -Null, 2- Low, 3 - Medium, 4 - High
IB_5	Knowledge Factors - Lack of information about markets	Single choice	1 -Null, 2- Low, 3 - Medium, 4 - High
IB_6	Knowledge Factors - Difficulties in finding cooperation from others to innovate	Single choice	1 -Null, 2- Low, 3 - Medium, 4 - High
IB_7	Market factors - Market dominated by established companies	Single choice	1 -Null, 2- Low, 3 - Medium, 4 - High
IB_8	Market factors - Uncertainty regarding the demand for innovative services	Single choice	1 -Null, 2- Low, 3 - Medium, 4 - High
IB_9	Reasons not to innovate - It is not necessary due to previous innovations	Single choice	1 -Null, 2- Low, 3 - Medium, 4 - High
IB_10	Reasons not to innovate - It is not necessary, because there is no demand for innovations	Single choice	1 -Null, 2- Low, 3 - Medium, 4 - High
FR_1	Financing by own means	Single choice	0 - No, 1 - Yes

## 2 Experimental design, materials, and methods

### 2.1 Experimental design

The data were obtained from businesses that conduct their hotel operations and services in the three cantons in the province of Santa Elena, located in the eastern region of the Republic of Ecuador. These enterprises are also registered in the Provincial Department of Tourism of Santa Elena. The fact that they are registered ensures that the objects of study are legally considered hotel establishments and offer the services corresponding to said activity, thus guaranteeing the quality of the information. The researchers established a final sample of informants representing 146 hotels.

The data were compiled using a structured questionnaire created by the authors which is based on the criteria of the Organization for Economic Cooperation and Development – OECD on organizational innovation [9], [10]. The questionnaire was designed to obtain information from the hotels and identify the factors which determine the activities of organizational innovation.

The sections and variables are the following:

1) Internal factors (IF), which correspond to the individual characteristics and internal structure of the business. It is composed by 6 factors: type of organization, years in operation, size of the enterprise, collaboration networks, training, and education level of administrators and organizational aspects. All these features have been defined in previous studies [1], [9], [11], [12], [13], [14]. Items for a type of organization (TYPE_1), years in operation (AGE_1), size of the enterprise (SIZE_1) and collaboration networks (NET_1) and were measured using a semantic differential type scale of an option. For training and education level of administrators, three variables (TRA_1–3) of semantic differential type scale of an option are used. Finally, for organizational aspects, it is analyzed in five variables (OA_1–5) on a scale of 4 points ranging from 1 “Not applicable” and 4 “High".
2) External factors (EF), beyond hotel business activity: obstacle impeding innovation and access to financial resources, presented in related studies [9], [11], [15], [16]. For the first one, Innovation Barriers is analyzed in 10 dimensions (IB_1–10) on a scale of 4 points ranging from 1 “Not applicable” and 4 “High”. The second component regarding access to financial resources was consulted on whether the financing is by its own means (FR_1) on a scale of 2 points (0 “No” and 1 “Yes").
3) The independent variable corresponds to organizational innovation, which is structured according to three items related to the methods of new practices organization (OI_1), Organization new methods (OI_2) and Management new methods (OI_3); all of which matches with the OECD guidelines [3]. These items are on a scale of 2 points (0 “No” and 1 “Yes").

For the first one, it is related to new practices in the organization of work or in the procedures of the company, this includes management in knowledge systems, reengineering, education system among others. The second, is related to the new methods of organizing jobs, to improve decision making, which includes management of work teams, restructuring of departments or implementation of responsibilities. Finally, new methods of managing external relations with other companies or institutions.

## 3 Materials

The data set was compiled by means of a survey addressed to the persons in charge of the management of the company. The data is available in Excel format, in two sheets. The first contains information on the variables, sections, labels and response options. The second sheet contains the responses of the 146 informants.

## 4 Methods

Researchers for the definition of the target population used the database of the Provincial Directorate of Tourism of the Province of Santa Elena, as the government regulator of tourism and hotel activity. This base is called the Tourist Cadastre, which consists of tourism businesses in the sector, including those that meet the requirements of infrastructure, personnel, equipment, and services to operate legally as a hotel. The geographical sector chosen for the research is homogeneous by sharing a space of regional political division, cultural and legal aspects, as well as being a sector of national and international tourist interest. Random sampling, obtaining a sample size of 146 hotels, carried out the selection process of the hotels in the research sector.

The authors as knowledgeable about the research topic made previous contacts with the hotel managers and experts in the subject, to carry out a pilot test of the semi-structured survey, which allowed establishing the content validity, construct validity and expert validity of the scale.

The research uses owners, general managers, and administrative assistants as key informants since they are considered as an important source of information related to the different variables that are consulted about the generation of organizational innovation in the company. The informants were informed about the confidentiality of the answers provided and that the aggregate level of analysis prevents the identification of the businesses or respondents, as well as the results to be presented at the level of academic publication.

**Fig. 1:**
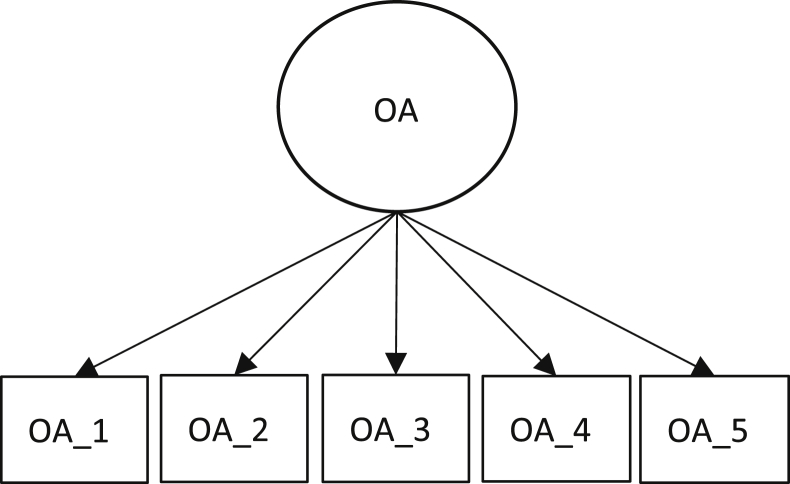
Organizational aspects model.

**Fig. 2:**
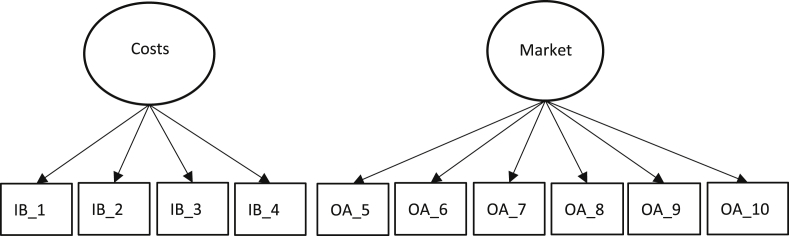
Innovation barriers model.

In the statistical analysis, the missing values were corrected, since they were within reasonable limits (less than 5% per indicator), the average replacement was used. An application of some variables within the scale and at the same time measure the reliability and validity of this, two models were established (see Fig. 1, Fig. 2). The first one explains the unidimensionality of the factor called Organizational Aspects and the second one, which tries to explain the organizational innovation barriers of the research sector.

Table 4 shows the factors related to Organizational Aspects (OA_1 to OA_5) and Innovation Barriers (BI_1 to BI_10), in which it can be seen that Cronbach's Alpha coefficient levels exceed the 0.7 limits, an adequate level of reliability [17]. Regarding the composite reliability (CR), it exceeds the 0.7 thresholds, revealing an adequate level of reliability [17]. On the other hand, the factorial loads are high and significant, which indicates a good convergent validity, despite having relatively low AEV values, possibly due to some factorial loads around 0.6.

**Table 4:** Reliability, internal consistency and convergent validity of the measuring instrument.

Dimension	Label	λ	CA	CR	AEV
**Organizational aspects**	OA_1	0.838**	0.88	0.91	0.67
	OA_2	0.804**			
	OA_3	0.841**			
	OA_4	0.784**			
	OA_5	0.836**			
**Innovation Barriers**					
Innovation Barriers - Costs	IB_1	0.613**	0.75	0.70	0.38
	IB_2	0.77**			
	IB_3	0.552**			
	IB_4	0.486**			
Innovation Barriers - Market	IB_5	0.612**	0.85	0.82	0.43
	IB_6	0.625**			
	IB_7	0.666**			
	IB_8	0.698**			
	IB_9	0.603**			
	IB_10	0.700**			

**p < 0.01; CA= Cronbach's alpha; CR = Composite reliability; AEV = Average extracted variance.

In an exploratory way and to understand the explanatory value of the data, a regression analysis was performed on the dependent variable OI including as independent variables AGE, SIZE, NET_1 and the variables resulting from the factor analyses of OA, IB_C (Innovation Barriers – Costs), and IB_M (Innovation Barriers – Market). The results are shown in Table 5 shows how the variables AGE, NET_1 and OA show a significant relationship with the dependent variable. It is necessary to mention that the VIF values are close to one, therefore it does not present multicollinearity.

**Table 5:** Regressions on variable “organizational innovation” (OI).

Variables	Unstandardized coefficient	VIF
AGE	−0.232*	1.029
SIZE	0.110	1.052
NET_1	0.373***	1.068
OA	0.146**	1.095
IB_C	0.005	1.495
IB_M	0.049	1.541
R^2^	**0.168**	

VIF = Variance inflation factor, ***p_value < 0,01 **p_value < 0,05 * p_value < 0,1.
