# Supplementary material for: Dataset on organizational innovation and its determinants in the SMEs hotels
Source: Data Brief. 2019 Aug 10;26:104352. doi: 10.1016/j.dib.2019.104352 (PMC6727002; doi:10.1016/j.dib.2019.104352)
Supplement: Multimedia component 1 [file mmc1.zip › Survey.pdf]

ORGANIZATIONAL INNOVATION FACTORS OF THE HOTEL SECTOR
ECUADOR (SANTA ELENA)

A. General information of the firm

A.1 Type of company

According to your commercial activity, indicate to which of the following groups you belong:

☐ Special Taxpayer ☐ Natural person not obliged to keep accounts
☐ Limited Company ☐ Natural person obliged to keep accounts
☐ Anonymous society

A.2 Age of the firm

Indicate the number of years the firm has

☐ Less than 5 years ☐ More than 5 years

A.3 Size of the firm

Indicate the number of employees who are working in the firm

☐ Micro firm (Less than 10 employees) ☐ Medium firm (51 to 250 employees)
☐ Small firm (10 to 50 employees)

A.4 Collaboration networks

The company is a member, partner or participant in Associations, Chambers or Business Organizations:

☐ Yes ☐ No

B. Details of the Manager and/or Owner

B.1 Position in the firm

Mention the position that you occupy in the company

☐ Owner ☐ Manager - Owner
☐ General Manager ☐ Administrative Assistant

B.2 Work Experience

The work experience you have in similar businesses is:

☐ Less than 1 year ☐ 2 to 3 years ☐ 3 to 5 years ☐ 5 or more years

B.3 Educational training

Indicate your educational training, which should be completed

☐ Elemental education ☐ Technological/technical ☐ Master degree
☐ High School ☐ University degree

C.Organizational Innovations

C.1 Introduction of organizational innovations by the administration

	Yes	No
New practices in the organization of work or in the procedures of the company	☐	☐
New methods organization of jobs, to improve decision making	☐	☐
New methods of managing external relations with other companies or Institutions	☐	☐

C.2 Importance of organizational innovations introduced

The following statements, rate them between high, medium, low or null:	High	Medium	Low	Null
Answer to needs of a client	☐	☐	☐	☐
Improvement of the ability to develop new processes	☐	☐	☐	☐
Higher quality of its services	☐	☐	☐	☐
Lower costs per unit of production of the service	☐	☐	☐	☐
Improvement in the exchange of information or communication within the company	☐	☐	☐	☐

D. Innovation activities in the company

D.1 Innovation Barriers

The following statements, rate them between high, medium, low or null:	High	Medium	Low	Null
Cost Factors - Lack of funds in the company	☐	☐	☐	☐
Cost Factors - Lack of financing for the company	☐	☐	☐	☐
Cost Factors - Innovation has a high cost	☐	☐	☐	☐
Cost Factors - Lack of qualified personnel	☐	☐	☐	☐
Market factors - Lack of information about markets	☐	☐	☐	☐
Market factors - Difficulties in finding cooperation from others to innovate	☐	☐	☐	☐
Market factors - Market dominated by established companies	☐	☐	☐	☐
Market factors - Uncertainty regarding the demand for innovative services	☐	☐	☐	☐
Market factors - It is not necessary due to previous innovations	☐	☐	☐	☐
Market factors - It is not necessary, because there is no demand for innovations	☐	☐	☐	☐

E. Economic situation of the company

The following statements, rate:	Yes	No
Financing by own means	☐	☐

We appreciate your help in answering this survey
